# Hirschsprung disease managed with one-stage transanal endorectal pullthrough in a low-resource setting without frozen section

**DOI:** 10.1186/s12893-022-01536-9

**Published:** 2022-03-08

**Authors:** Samuel Negash, Hanna Getachew, Dagnachew Tamirat, Tihitena Negussie Mammo

**Affiliations:** 1 Unit of Pediatric Surgery, Addis Ababa University, Addis Ababa, Ethiopia; 2 Department of Pathology, Addis Ababa University, Addis Ababa, Ethiopia

**Keywords:** Transanal endorectal pullthrough, Frozen section, Hirschsprung’s disease, Ethiopia

## Abstract

**Background:**

Over the past few decades, surgery for Hirschsprung’s disease (HD) has evolved into a minimally invasive, single-stage procedure with excellent outcomes. Intraoperative frozen section biopsy is critical for this procedure to avoid the potential risk of leaving a retained aganglionic segment. However, this facility is not available in most low-income countries. Therefore, a two-stage procedure with an initial colostomy is still practiced in the developing world. We aimed to evaluate the outcome of single-stage transanal pullthrough performed in a facility without frozen section biopsy.

**Methods:**

A retrospective review of all patients who underwent transanal pullthrough in two teaching hospitals over a 6-year period (2015–2020).

**Results:**

Forty-seven children underwent transanal endorectal pullthrough (TERPT). Age at surgery ranged from 2 months to 6 years and mean weight was 8.7 kg. Barium enema did not show transition zone in 6 patients (12.8%) while others demonstrated short segment HD. Intraoperatively, the transition zone was visualized in 40 patients (85%). TERPT alone was performed in 35 (74.5%), TERPT with laparotomy to visualize transition zone in 9 (26.7%) and TERPT with transabdominal mobilization was required in 3 (6.4%). Definitive histopathologic examination revealed aganglionic segment pullthrough in 4 (8.5%) and transitional zone pullthrough in another 4 (8.5%). However, with long term follow up all eight children remained asymptomatic and no intervention was required.

**Conclusions:**

Transanal pullthrough offers reduced number of surgeries and faster recovery. We have also observed a good functional outcome despite a discrepancy with pathology results. Overall, our data suggests it is a safe and viable option for the treatment of short segment HD in facilities where frozen section is not available.

## Introduction

Hirschsprung’s disease is one of the most common causes of intestinal obstruction in children [1, 2]. Short segment HD accounts for 80% of the cases [3]. Traditionally, this condition use to be managed with a 2-stage surgery. Initially a proximal diverting colostomy was done followed by transabdominal pull-through after a couple of months [4]. In 1998, there was a significant shift in the approach with the discovery of the single stage transanal pull-through procedure [5, 6].

Many surgeons abandoned the routine use of a colostomy in favor of this procedure [7]. Subsequently, studies have shown it to be safe, even in newborns, and it has become the standard of practice in many centers [8, 9]. This procedure became popular because of the simplified nature and potential for cost savings [10]. It is also a minimally invasive technique that offers faster recovery with fewer complications [11, 12]. All these qualities are extremely attractive for low income countries where there are few pediatric surgery centers with limited resources [13].

However, the procedure has one drawback. It is dependent on frozen section for identification of the normally innervated bowel [6]. Frozen section is described as a critical part of the procedure because barium enema may be inaccurate in 10% of the cases [14, 15]. Without frozen section confirmation, there is a potential risk of leaving aganglionic bowel behind in these patients [15].

Frozen section facility is not available in most LMIC such as ours, thus mandating colostomy for all children with this condition. Nonetheless, we recently started performing single stage pullthrough in selected patients despite the lack of this facility. We aim to report our initial experience and to determine whether this is a possible alternative for other resource-limited countries.

## Methodology

We conducted a retrospective hospital-based study at Tikur Anbessa Hospital and Menelik Hospital. These are the largest centers affiliated with Addis Ababa University, Ethiopia. They have a high burden of pediatric surgical cases, with more than 2000 procedures being performed yearly.

We included all children less than 15 years of age who underwent one stage transanal pullthrough over the past 6 years (January 2015–December 2020). Data was obtained from medical records using a structured questionnaire. Variables included demography, symptoms, operative details, post-op complications and histopathology result. Telephone interviews were also conducted for follow-up data on July 2021. Data was then entered, coded and analyzed using SPSS version 23. Ethical approval was obtained from the institutional review board of the college of health sciences, Addis Ababa University.

The single-stage transanal pullthrough procedure was offered to selected patients with HD in our institution. The considerations for selecting these patients were

1. Hirschsprung disease confirmed with full thickness rectal biopsy
2. Short segment disease without significant dilatation of the descending colon (diagnosed on barium enema)
3. No severe enterocolitis or severe malnutrition
4. Respond to rectal irrigation until the age of 3 months or weight of 5 kg (surgery delayed in order to avoid anesthesia-related complications.)

The soave pullthrough technique was used in all cases. Extent of resection was determined by barium enema and intraoperative visualization of transition zone by the surgeon. Dilated bowel above transition zone was resected as much as possible until normal caliber bowel was reached. Biopsy (for permanent section) was taken from proximal pulled down segment.

## Results

Over a period of 6 years, 47 children underwent transanal soave pullthrough. There were 39 boys (83%) and 8 girls (17%). Half of the children were from Addis Ababa (51.1%) while others came from different regions in the country. Mean follow up time was 11.5 months.

Thirty-eight children (80.9%) had symptoms since birth. Presenting complaints were chronic constipation in 39 children (83%), acute obstruction in 5 (10.6%) and 3 children (6.4%) had pseudo-incontinence. Only one child had malnutrition and anemia which was corrected before the surgery.

**Fig. 1:**
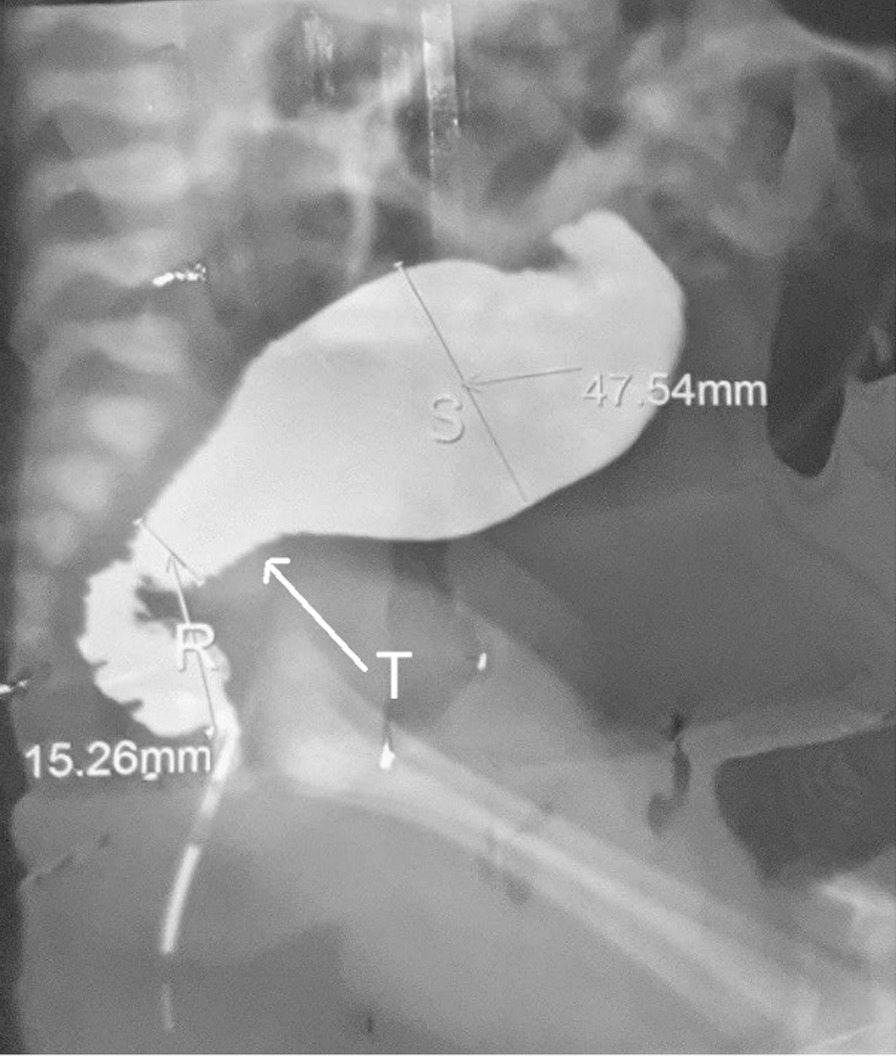
Barium enema of one of the participants showing classic features of HD with narrow rectum (R), dilated sigmoid (S) and funnel shaped transition zone (T)

Age at surgery ranged from 2 months to 6 years (median 6 months) and mean weight was 8.7 Kg. Barium enema did not show transition zone in 6 patients (12.8%) while others demonstrated short segment HD (Fig. 1). Intraoperatively, transition zone was visualized in 40 patients (85%). Transition zone was in rectum in 2 (4.3%), rectosigmoid in 26 (55.3%) and sigmoid in 12 (25.5%).

**Fig. 2:**
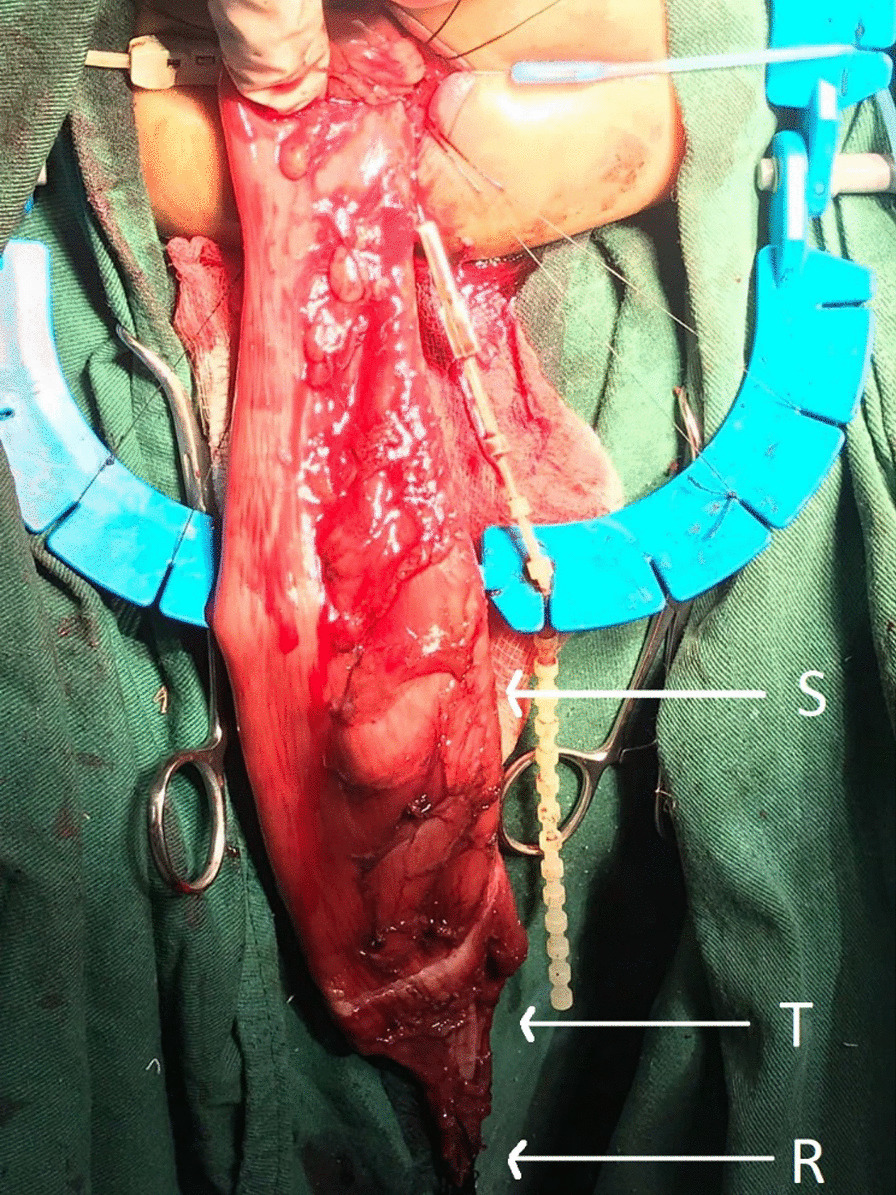
Intraoperative picture of one of our cases of TERPT demonstrating rectum (R), dilated sigmoid (S) and transition zone (T). Note that resection has proceeded proximal to the dilated sigmoid until normal caliber bowel is reached

TERPT alone was performed in 35 patients (74.5%) (Fig. 2). In 9 patients (26.7%) a small left lower quadrant incision was made to visualize the transition zone before starting TERPT. Laparotomy for transabdominal mobilization was required in 3 patients (5.4%).

The mean operating time was 118.9 minutes. There were no intraoperative anesthetic complications. Intraoperative transfusion was required in 2 children (4.3%). Post-operative hospital stays ranged from 2 days to 1 month (median 4 days). Resumption of oral feeding ranged from 1 to 6 days (mean 2 days).

**Fig. 3:**
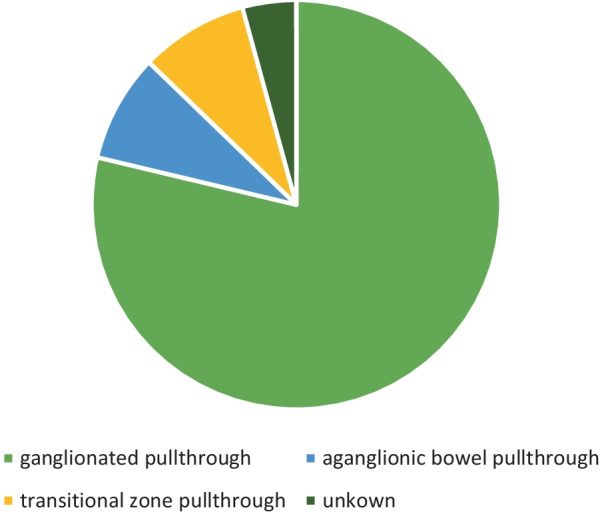
Definitive histopathology results after pullthrough performed without frozen section confirmation

Definitive histopathologic examination (permanent section) revealed aganglionic segment pullthrough in 4 (8.5%) and transitional zone pullthrough in another 4 (8.5%). All eight children remained asymptomatic and no intervention was undertaken. The children also did not require laxatives or enemas. In 2 of the children (4.2%) the bowel status is unknown because biopsy sample was lost. (Fig. 3)

Post op complications occurred in 25 children (53.2%). The most common complication was perineal excoriation which occurred in 13 children (27.7%). Other complications are outlined in Table 1. Two of the children with abdominal incision had wound infection. One resolved with antibiotics but the other developed wound dehiscence that required secondary closure.

**Table 1:** Postoperative complications

Post-op complications	N (%)
Perianal excoriation	13 (27.7%)
Wound infection	2 (4.3%)
Enterocolitis	5 (10.6%)
- Death	1
Obstructive symptoms	4 (8.5%)
- Twisted pullthrough	1
- Acquired aganglionosis	1
- Suspected internal sphincter achalasia	2
Constipation	3 (6.3%)
Anastomotic leak	1 (2.1%)

There was one late death 10 days after discharge due to enterocolitis. The child died during transportation before the parents could get back to our hospital.

Three children required redo pull thorough (6.4%). All three children had ganglionated pullthrough. The indications for redo operation in the three children were twisted pullthrough, acquired aganglionosis and anastomotic leak. All children underwent a colostomy before the redo-pullthrough.

Continence was assessed in those over the age of 3 years which were 28 (59.6%). Of these, 5 (17.8%) report streaking on underpants and 2 (7.1%) report occasional soiling. Both children who had postoperative soiling accidents, were the children who had soiling pre-operatively and have noted improvement. There were no children with overt incontinence.

## Discussion

It is proven that TERPT has several advantages over the two stage pullthrough procedure as it offers reduced number of surgeries, faster recovery and overall reduced cost [10, 12]. Our data also substantiates this finding as we found less operating time (118 min), early resumption of oral feeding (2 days) and early hospital discharge (5 days) comparable to other literatures on TERPT [16, 17]. These factors are especially important low income settings as there are limited resources. Moreover, this procedure avoids a colostomy which has a high psychosocial burden in the developing world [18, 19].

Intraoperative frozen section is considered a standard part of the procedure [6, 15] and we faced ethical concern in performing one stage TERPT without this facility. However, studies on the pathology of HD have shown that even intraoperative frozen section can have a discrepancy with permanent section in 3–15% [20–22]. We also considered frozen section may be less reliable in developing nations as it is dependent on availability of experienced pathologist [20].

Additionally, our practice was supported by other studies that reported contrast enema and/or intraoperative naked eye visualization are sufficient for the identification of transition zone [1, 3, 7, 10, 16, 23] (Table 2). Some of these authors also recommend resecting normal appearing bowel more proximal to transition zone to ensure pullthrough of ganglionated bowel [7, 16]. This was the practice in our setting as well.

**Table 2:** Comparison of studies on single stage pullthorugh performed without frozen section

	Study	Country	No of patients	Type of PT	Length of resection (mean)	Aganglionic PT	Aganglionic PT requiring redo
1	Sookpotarom (2009) [1]	Thailand	27	Swenson	16 cm	4	0
2	Khalaf (2014) [10]	Iraq	6	Swenson	NA	0	0
3	Choudhury (2014) [16]	Bangladesh	31	Soave	22 cm	0	0
4	Shrestha, (2014) [3]	Nepal	20	Soave	25 cm	1	1
5	Ahmed (2017) [7]	Sudan	15	NA	30 cm	NA	NA
6	Chawngthu (2018) [23]	India	21	Swenson	19 cm	0	0
7	Negash, 2021 (this study)	Ethiopia	48	Soave	NA	4	0

Retained aganglionosis occurred in 3 of the 7 studies who performed single stage transanal pullthrough without frozen section. The study by Shrestha [3] reported 1 out of 20 cases which is an acceptable rate. They performed redo pullthrough because of the biopsy result. Significant rate of retained aganglionosis occurred in the study by Sookpotarom (14.8%) [1] followed by our current study (8.5%). In both studies however, the patients did not have symptoms and did not undergo intervention. We deferred redo-surgery because we chose to make decisions based on clinical symptoms rather than relying on pathology results.

Although uncommon, late diagnosed Hirschsprung can present with soiling preoperatively [24]. We had two such patients who persisted to have some soiling after the procedure. Otherwise, good continence was reported from a majority of the patients which is expected [25]. Other complications encountered in our study include wound infection, enterocolitis, anastomotic leak, twisted pullthrough, which have comparable rates to other studies [15, 26, 27]. These complications are also not specific to one stage TERPT and can occur in the two stage pullthrough procedure [28].

## Conclusion

Our experience, as well as literature reviewed, has demonstrated the feasibility of performing transanal pullthrough in the absence of frozen section facility. It had acceptable short term and long-term outcomes with none of the complications being attributed to remnant aganglionic segment. This procedure should serve as an alternative in the treatment of HD in children from LMIC.

## Data Availability

The datasets used and/or analyzed during the current study available from the corresponding author on reasonable request.

## Abbreviations

HD: Hirschsprung’s disease
LMIC: Low-income and Middle-income countries
TERPT: Transanal endorectal pullthrough
PT: Pullthrough
